# A Digital Box and Block Test for Hand Dexterity Measurement: Instrument Validation Study

**DOI:** 10.2196/50474

**Published:** 2023-09-15

**Authors:** Eveline Prochaska, Elske Ammenwerth

**Affiliations:** 1 Institute of Applied Electronics and Technical Informatics University of Applied Sciences Campus Vienna Vienna Austria; 2 Institute of Medical Informatics Private University for Health Sciences and Health Technology Hall in Tirol Austria

**Keywords:** assessment, Box and Block Test, BBT, concurrent validity, dexterity, digital Box and Block Test, dBBT, hand dexterity assessment, interrater reliability, test-retest reliability, validate, validity

## Abstract

**Background:**

The Box and Block Test (BBT) measures unilateral gross manual dexterity and is widely used in clinical settings with a wide range of populations, including older people and clients with neurological disorders.

**Objective:**

In this study, we present a newly developed digitized version of the BBT, called the digital BBT (dBBT). The physical design is similar to the original BBT, but the dBBT contains digital electronics that automate the test procedure, timing, and score measurement. The aim of this study is to investigate the validity and reliability of the dBBT.

**Methods:**

We performed measurements at 2 time points for 29 healthy participants. BBT and dBBT were used at the first measurement time point, and dBBT was used again at the second measurement time point. Concurrent validity was assessed using the correlation between BBT and dBBT, the paired *t* test, and the Bland-Altman analysis. Test-retest reliability and interrater reliability were examined using the interclass correlation coefficient (ICC) by repeated measures with the dBBT within an interval of 10 days.

**Results:**

Our results showed moderate concurrent validity (*r*=0.48, *P*=.008), moderate test-retest reliability (ICC 0.72, *P*<.001), a standard error of measurement of 3.1 blocks, and the smallest detectable change at a 95% CI of 8.5 blocks. Interrater reliability was moderate with an ICC of 0.67 (*P*=.02). The Bland-Altman analysis showed sufficient accuracy of the dBBT in comparison with the conventional BBT.

**Conclusions:**

The dBBT can contribute to objectifying the measurement of gross hand dexterity without losing its important characteristics and is simple to implement.

## Introduction

Dexterity is the ability of a person to use their fingers, hands, and arms to perform tasks such as activities of daily living [1]. Manual dexterity is an important indicator of upper limb motor function [2] and is frequently measured by researchers and clinicians to represent rehabilitative effectiveness [3]. One of the most commonly used assessments for gross manual dexterity (International Classification of Functioning, Disability and Health domain mobility d4) is the Box and Block Test (BBT) [4,5]. The BBT is easy to understand, requires a short time to complete, and is suitable for individuals with limited hand function. In addition to gross dexterity, the BBT assesses other motor components, such as eye-hand coordination or crossing the partition wall [6]. Furthermore, a strong correlation has been found between the BBT and activities of daily living [7]. The BBT consists of a box divided into 2 equal parts by a partition. The task requires transporting blocks from 1 box to another at a time. The result of this test is the number of transported blocks with 1 hand within 60 seconds. The BBT was validated with healthy people. The resulting scores are then compared with clinical norm data [8,9]. Benefits of the BBT include ease and speed of implementation.

Despite the seemingly simple determination of the final result (number of blocks in 60 seconds), the therapist must observe carefully during the execution of the test—in addition to timing with a stopwatch—to detect possible errors in the execution. Care must also be taken to ensure that the patient moves only 1 block at a time from 1 box to another. If several are transported at the same time, only 1 block is added to the result. When transporting the individual block, the participant must cross the partition of the BBT with their fingers at a time, and this must also be monitored. Thus, the errors detected by observation minimize the final result, which could affect the reliability and objectivity of the evaluation. By automating the timing and correct counting of the blocks, a possible variability of the evaluation should be minimized, which ensures comparable test results over time. The BBT assesses a change in hand function over time. The automation of the test sequence minimizes possible variances due to different testers.

Several further developments use different technologies in addition to the conventional BBT to increase the objectivity and reliability of the manual dexterity measurement. Using the traditional BBT, various technologies have been used to digitally capture hand movement during test execution, such as depth cameras [10], motion sensors [11], or infrared sensors [12]. Furthermore, there are several research works using virtual reality [13-16]. All these developments have in common that the easy handling of the conventional BBT is lost, as a considerable amount of equipment is required and therefore technical understanding from users. At the same time, data collection is automated and improved.

We have thus developed a digital version of the BBT—the digital Box and Block Test (dBBT)—that combines the advantages of automatic data collection with ease of use. The aim of this study was to validate the dBBT in comparison with the original BBT in healthy adults. In particular, this study aimed to evaluate: (1) the concurrent validity, (2) the test-retest reliability, and (3) the interrater reliability of the dBBT.

## Methods

### Overview

We follow the COSMIN (Consensus-Based Standards for the Selection of Health Status Measurement Instruments) standard, which is the consensus-based checklist for the preferred design characteristics and statistical methods of studies on measurement properties [17].

### Study Design

This research follows a test-retest design with crossover. The participants were randomly matched into 2 groups. Data for BBT and dBBT were collected at 2 measurement time points, with crossover after the first measurement point. The total data collection period was 10 days.

A total of 2 testers (raters 1 and 2) conducted all data collection. Before the study, the 2 testers performed 2 pretests.

### Conventional BBT

The BBT was developed by Jean Ayres and Patricia Buehler in 1957 and modified to the current version by Patricia Buehler and Elizabeth Fuchs in 1976. Normative data for children and adults were established in 1985 [8,9].

The BBT is a widely used outcome measure to quantify upper limb motor function, especially gross manual dexterity [6]. The BBT comprises a wooden box (53.7 cm×25.4 cm×8.5 cm) that is divided into 2 compartments (25.4 cm each) by a partition and 150 blocks (cubes with 2.5 cm side length) in 1 of the 2 boxes [9]. Participants have to move the blocks one by one from 1 compartment of a box to another in 60 seconds. The BBT is timed with a stopwatch, and after 60 seconds, the transported blocks (on average 75-90 for healthy persons) are to be counted by the test administrator. A 15-second trial period is permitted at the beginning of the test.

### The dBBT

**Figure 1:**
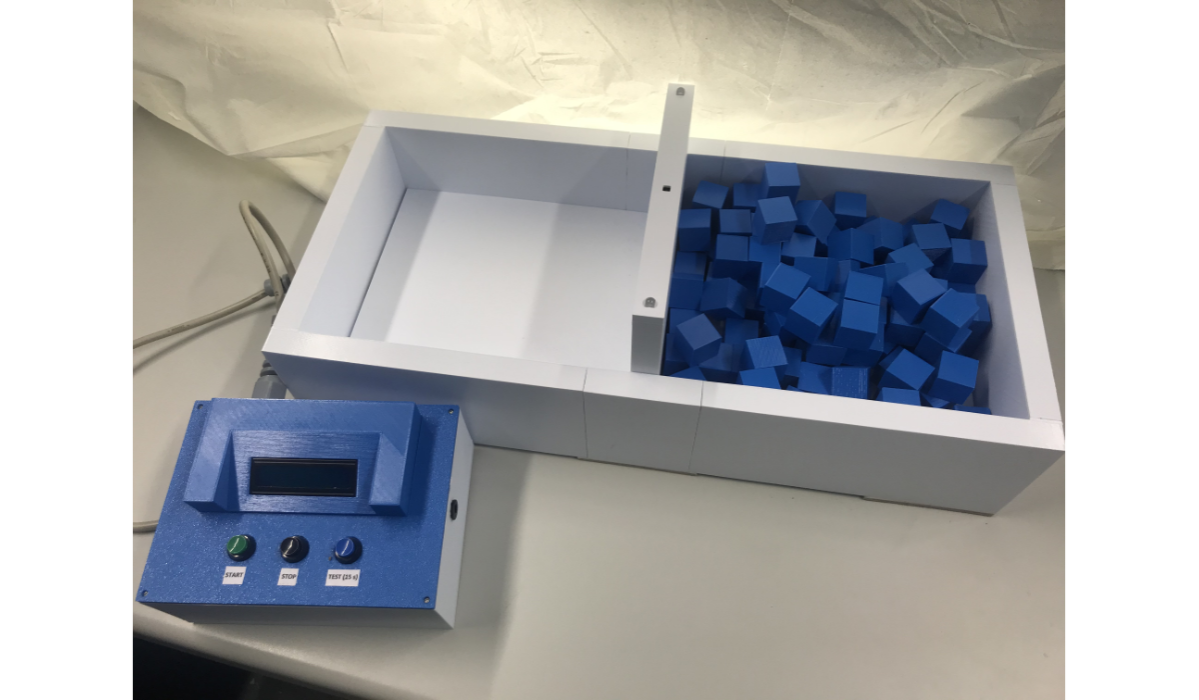
The components of the digital Box and Block Test board and control unit.

The digital version of the BBT, the dBBT, is quite similar to the BBT but uses digital measurements. We have developed the dBBT to further standardize the measurement with the BBT by using digital functions to automatically measure the time and the achieved scores. Figure 1 shows an overview of the dBBT. The dBBT consists of the control unit and the test box with a partition. The test box is in form and dimensions oriented to the specifications of Mathiowetz et al [9]. The dBBT and the blocks were created using a 3D printer. Load cells are installed in the bottom of the 2 boxes to record the number of blocks automatically. A microcontroller in the control unit processes the sensor signals, automatically measuring the test time, and controls the user inputs through the buttons and the output through the display.

On the control unit, the start button starts the timing, and the LEDs on the partition light up green until the test time is over; then they light up red. The dBBT automatically counts the valid blocks (if 2 blocks are transported at the same time, the system counts only 1 block for the valid result) and shows the achieved score (number of blocks in 60 seconds) on the display. Also, the 15-second trial period is provided by the dBBT.

The prototype of the dBBT enables the assessment according to the standardized specifications of Mathiowetz et al [9].

### Participants and Recruitment

Participants were occupational therapy students at the University of Applied Sciences in Vienna (Austria). The sample size calculation for evaluating correlation was calculated with G*Power Version 3.1.9.7 (Heinrich-Heine-University Düsseldorf). The calculation with the factors correlation point biserial model, 2 tails, effect size 0.5 [18,19], α error .05, and power 0.85 [20] resulted in a sample size of 26. Participants were recruited in the fall of 2022 through a presentation of the study in collaboration with a faculty member in the program. The inclusion criteria were (1) individuals without a history of neuromuscular or orthopedic dysfunction that would significantly affect dexterity and (2) 18 years or older. Handedness was identified by asking the participant which hand was used for writing. In total, 32 people participated in this study.

### Data Analysis

We used SPSS Statistics (version 28.0; IBM Corp) for data analysis. Descriptive statistics were used to describe the study population. The normality of the data was evaluated using the Shapiro-Wilk test. Concurrent validity was determined by the Pearson correlation coefficient (*r*) for the relationship between the conventional BBT and the dBBT at measurement point 1. The correlation was classified as follows: no or very low, *r*=0-0.25; low, *r*=0.26-0.40; moderate, *r*=0.41-0.69; high, *r*=0.70-0.89; and very high, *r*=0.90-1.0 [20]. The level of statistical significance was set at *P*≤.05.

The agreement between the BBT and dBBT was examined using the Bland-Altman analysis to check for systematic bias and estimate the limit of agreement (LOA) [20,21]. In the Bland-Altman scatter plot, the x-axis represents the mean of these measurements, and the y-axis shows the difference between the 2 paired measurements. The fixed bias was statistically evaluated using the 95% CI of the mean differences between the BBT and dBBT values. A fixed bias is present when 0 is not within the range of the CI. After ensuring that the differences are normally distributed, SD can be used for defining the LOA mean (SD 1.96) [22]. LOAs show how much the scores can vary in stable patients. A change in scores within LOAs or smaller indicates a measurement error; outside the LOAs, it can be assumed that these are statistically significant changes [20].

For assessing interrater and test-retest reliability, intraclass correlation coefficients (ICC) were used. To estimate the correlation, the following classification of correlation was used [23]: less than 0.5 poor, between 0.5-0.75 moderate, between 0.75-0.9 good, and greater than 0.9 excellent. Measurement error was determined by estimating the standard error of measurement (SEM) using the formula 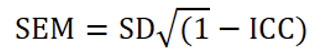, where SD is the standard deviation of the means from all probands [20] of the test-retest scores and ICC from the test-retest reliability. Smallest detectable change (SDC) was calculated, based on the test-retest parameter SEM, as follows: 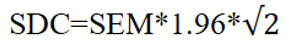 [20]. The SDC represents an absolute measure of reliability (measurement error) and is used to assist in interpreting results and determining whether a change between repeated tests is a random variation or a true change in performance [24].

### Data Exclusion

In the data set, outliers became apparent after data collection during the initial data analysis. These outliers showed up in differences in the measurement repetitions. Values with more than 20% (above the 90th percentile) difference between 2 measurements cannot be assigned to any natural variance in healthy persons. As the participants were all individuals with unrestricted hand function, a true outlier can be ruled out. A possible reason is seen as an error in the test execution or data collection. Therefore, 3 corresponding data sets from a total of 32 participants were excluded from further analysis. The sample size of the assessed data was thus 29.

### Experimental Procedure

The study design includes 2 measurement time points. The test procedures took place in a room specially prepared for this purpose at the University of Applied Sciences Campus Vienna. The setting and test instructions for the BBT and the dBBT corresponded to the standard set by Mathiowetz et al [9]. The test instructions were translated into German by the author. One measurement of the writing hand of each participant was performed. Participants sat on a chair in front of a table. The test box was centrally located in front of them. The box with the blocks was on the side of the hand to be tested. The instructions for the test were read out by the tester according to the standardized instructions, including a short demonstration. The participants performed a 15-second trial period before the recorded test. For the start, the participants have to position their hands on the left and right sides of the box; then the start signal is given, and the timing starts [9]. The tests were timed at the BBT with a stopwatch and at the dBBT with the implemented time measurement at the push of a button. If the participant transports several blocks at the same time, only one is counted. If a block has fallen from the table, the participant should not be distracted by it and continue with the task. If the block was already transported over the partition before it fell down, it will be counted in the result [9].

Data collection took place at 2 measurement times, with 10 days in between. This period was chosen to be small enough so that no change in hand function occurs, but at the same time large enough to minimize influences from practice or memory [18,25]. A total of 29 participants were randomized into both groups, resulting in 14 participants in group 1 and 15 in group 2. At the first measurement, group 1 was tested from tester 1 with the dBBT, and then on the same day using the conventional BBT. Group 2 was tested by tester 2 in reverse order (first the conventional BBT, and then the dBBT).

At the second measurement point, 10 days after the first measurement, a total of 15 participants took part. Both groups were tested using the dBBT. Here, both groups changed the tester: group 1 was thus tested by tester 2 and group 2 by tester 1.

This study design was chosen to allow assessing both test-retest reliability and interrater reliability as well as the validity of the dBBT compared with the BBT.

### Ethics Approval

The study protocol was in accordance with the Declaration of Helsinki and was approved by the ethics committee (EK Nr 97/2022) of the University of Applied Sciences Campus Vienna. This study has been registered on the Open Science Framework [26].

## Results

### Participant Characteristics

The characteristics of the healthy participants who participated in this study are summarized in Table 1. The mean age of the participants was 23.5 (SD 5.2) years. The majority of the participants (n=28) were female and right-handed.

The second measurement point was completed by 15 probands. Table 2 shows the means, SDs, maximum and minimum scores, and the number of valid values of the 3 measurements with the BBT, dBBT1 (both at the first measurement time point), and dBBT2 (at the second measurement time point). The BBT shows on average a few higher scores than the dBBT1 and dBBT2. The average score ranges (blocks in 60 seconds) are 81.83 for the BBT, 76.86 for the dBBT1, and 80.71 for the dBBT2.

**Table 1:** Participant characteristics (N=29).

Characteristics		Value
Sex (female), n (%)		28 (97)
Age (years), mean (SD)		23.5 (5.2)
**Tested hand, n (%)**		
	Right	28 (97)
	Left	1 (4)

**Table 2:** Average performance of healthy persons taking the Box and Block Test (BBT) and the digital BBT (dBBT) (blocks in 60 seconds).

	Score, mean (SD)	Score, range	Valid values
BBT^a^	81.83 (6.35)	69-93	29
dBBT1^b^	76.86 (4.98)	68-86	29
dBBT2^c^	80.71 (7.75)	63-92	15

^a^Scores of original BBT at time point 1.

^b^Scores of dBBT at time point 1.

^c^Scores of dBBT at time point 2.

### Concurrent Validity

A Pearson correlation analysis was performed to determine if there was a correlation between the variables BBT and dBBT1 and between BBT and dBBT2.

Our examination of BBT and dBBT1 (n=29) showed that BBT had higher scores (mean 81.83, SD 6.35) than dBBT1 (mean 76.86, SD 4.98) and dBBT2 (mean 80.71, SD 7.75). There was a moderate correlation of *r*=0.48 between the variables BBT and dBBT1. The result of the Pearson correlation analysis showed that there was a significant relationship between BBT and dBBT1 (*r*_29_=0.48, *P*=.008).

A dependent samples *t* test showed that the difference between the scores of BBT and dBBT1 was statistically significant (*t*_28_=−4.96, *P*<.001; 95% CI −7.21 to −2.72).

**Figure 2:**
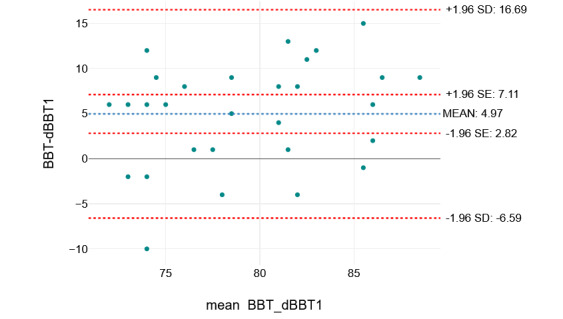
Bland-Altman plot for agreement between the scores of the Box and Block Test (BBT) and the digital BBT (dBBT1).

The Bland-Altman plot to evaluate the agreement between BBT and dBBT1 is shown in Figure 2. The fixed bias was statistically evaluated using the 95% CI (SE 1.96) of the mean differences between the BBT and dBBT values. For BBT and dBBT1, the mean difference was 4.97 (7.11-2.82), and a fixed bias was present.

All obtained values of BBT and dBBT1 (except one) were in the range of the LOAs (16.69 to −6.59), which indicates a sufficient agreement between the 2 measurement methods.

### Test-Retest Reliability

For the calculation, the scores of the dBBT (blocks in 60 seconds) were compared at the 2 measurement points within a 10-day interval. From the whole sample of 29 healthy participants, 15 completed the second measurement. A total of 14 participants did not attend the second measurement point without giving a reason. The test-retest reliability for these 15 participants was determined by calculating the ICC (3,*k*) based on the 2-way mixed model (*k* fixed raters are defined), absolute agreement (agreement between 2 raters is of interest), and average measure [20,27]. The ICC is moderate, with an ICC of 0.72 (−0.23 to 0.93; *P*<.001). Because the ICC is only an expected value of the true ICC, it is appropriate to assess the degree of reliability on the basis of the 95% CI of the ICC value and not the ICC value itself [23]. The value 0 is included in the CI 95% range, indicating that the correlation is not statistically significant.

An SEM of 3.1 blocks was identified, which represented 3.88% of the mean score observed in the test-retest session.

The SDC was 8.5 blocks (10.77%); 95% of the tested population had a random variation of less than 8.5 blocks on repeated testing, and a value above would indicate a true change beyond an expected measurement error. An SDC% <10% is considered to indicate an excellent random measurement error [3]. The SDC% (10.77%) of the dBBT indicates that the dBBT is capable of supporting clinicians in assessing the significance of outcomes and interpreting treatment efficacy.

### Interrater Reliability

Interrater reliability was assessed with ICC (2,*k*), based on the 2-way mixed and consistency model [20,27]. For this purpose, the results of tester 1 and tester 2 were compared for the 15 participants who completed the dBBT at both measurement points. The calculated interrater reliability was moderate, with an ICC of 0.67 (0.02-0.89; *P*=.23) and was statistically significant. The result was close to the limit of high interrater reliability, which is 0.7 [18].

## Discussion

### General

The aim of this study was to evaluate the concurrent validity, the test-retest reliability, and the interrater reliability of the newly developed dBBT.

Previous studies have presented various further developments of BBT assessments, such as the conventional BBT extended with additional technologies [10-13,28]. Other works have also investigated the BBT using virtual reality [14-16,29]. Compared with these BBT implementations, our new dBBT is unique because no additional technical equipment is required. It is a stand-alone solution, like the conventional BBT, and therefore does not require any additional skills from the test administrator or proband. At the same time, it offers digital functions that support the execution of the measurement (collection of time and result).

### Concurrent Validity of the dBBT

On the whole, participants moved fewer blocks with the dBBT1 (mean 76.86, SD 4.98) and the dBBT2 (mean 80.71, SD 7.75) than in the original BBT (mean 81.83, SD 6.35).

The comparison of the new dBBT with the original BBT found a moderate correlation between the BBT and dBBT (*r*_29_=0.48, *P*=.008). These results are comparable to Everard et al [4], who reported a correlation of *r*=0.58 (*P*<.01) for healthy people who completed hand dexterity measurement with the BBT and a virtual reality version of the BBT.

The scores of dBBT1 were significantly lower than the scores of BBT measurements (*t*_28_=−4.96, *P*<.001; 95% CI −7.21 to −2.72).

The Bland-Altman plot showed that the dBBT1 achieved, on average, 4.97 fewer blocks than the measurement with the BBT. As the value 0 is not in the 95% CI of the mean (7.11-2.82), a fixed bias is assumed. All but one of the values collected fell within the LOAs (16.69 to −6.59), indicating that the dBBT has sufficient accuracy to provide an accurate measure of hand dexterity.

### Test-Retest Reliability of the dBBT

The test-retest reliability, ICC (3,*k*), of the 2 dBBT sessions (n=15) was moderate, with an ICC of 0.72 (−0.23 to 0.93; *P*<.001), in healthy adults. A comparable study by Everard et al [4] reported ICC values of 0.7 to 0.9.

The SEM calculated for the dBBT was 3 blocks. SDC, useful for interpretation of real changes in hand dexterity, was 8.5 blocks (10.77%) for dBBT.

The ICC value indicates what proportion of the total variance over a range of values is due to heterogeneity among study participants [30]. In this study, only healthy participants of mainly similar age were tested. A lack of variance among the participants may result in a lower ICC value [20].

### Interrater Reliability of the dBBT

The examination of interrater reliability showed a moderate ICC of 0.67 (0.02-0.89; *P*=.23). In contrast, in the study by Mathiowetz et al [9], a high interrater reliability (*r*=0.85-0.99) was reported. However, this study is not directly comparable because the calculations were made using the Pearson correlation coefficient, which is no longer considered contemporary [17,20]. Platz et al [31] also showed high interrater reliability with an ICC>0.9.

It should be noted in this interrater reliability result that the sample has low variances, which may lead to a low ICC value [20].

### Clinical Implications

The BBT is suitable for use in clinical settings. It measures the dexterity performance of the hand. The BBT is particularly recommended for progress measurements of patients with neurological disorders [32]. The BBT is mainly used to assess therapy effects, that is, a measurement is taken at the beginning of a defined period and a repetition at the end. The assessment of a possible therapy effect is solely based on the comparison of these 2 measurements. Therefore, the fact that the dBBT measures on average 5 blocks less than the original does not affect its suitability as a measurement tool. It does not affect the ability of the dBBT to assess a possible therapeutic effect.

The dBBT shows moderate results in test-retest and interrater reliability. The dBBT enables compliance with the standardized measurement protocol, according to Mathiowetz et al [9]. It automatically measures the test time, counts the transported blocks, and shows the achieved result on a display. These functions help to increase objectivity. The material (plastic) is well suited for clinical use, compared with the original, which is made of wood. The shape of the dBBT is similar to the BBT, so it is just as easy for clinicians to transport and use.

In the next step, the practicability of the dBBT will be investigated in qualitative studies in order to be able to make statements about its clinical utility. After that, studies are planned with populations that typically use the BBT, with people after stroke and people with multiple sclerosis. These steps, which follow this study, will make it possible to make statements about the generalizability of the results.

### Limitations

The study was conducted with healthy individuals without hand dexterity limitations. Therefore, the results need to be confirmed in future studies in patients with hand dexterity impairments.

In this study, the results of hand dexterity measurements from 2 measurement time points were collected and compared. From the authors’ point of view, the fact that the majority of the participants were female had no influence on the present results.

The sample size was calculated to be sufficient for group comparisons according to our power analysis. However, at the second measurement time point, only 15 people participated, which could affect the strength of the calculations for test-retest reliability and interrater reliability.

The homogeneity of the participant group could also have an influence on the results.

### Conclusions

This study showed that the newly developed dBBT is a valid, reliable, and usable tool to assess manual dexterity among healthy participants. The dBBT provides automatic timing and counting to help further objectify the results of hand dexterity measurement.

## Abbreviations

BBT: Box and Block Test
COSMIN: Consensus-Based Standards for the Selection of Health Status Measurement Instruments
dBBT: digital BBT
ICC: intraclass correlation coefficient
LOA: limit of agreement
SDC: Smallest Detectable Change
SEM: standard error of measurement

## Data Availability

The data that support the findings of this study are available on request from the corresponding author.

## References

[1] Oliveira CS, Almeida CS, Freitas LC, Santana R, Fernandes G, Junior PRF, Moura RCF (2020). Use of the box and block test for the evaluation of manual dexterity in individuals with central nervous system disorders: a systematic review. Man Ther Posturology Rehabil J.

[2] Desrosiers J, Bravo G, Hébert R, Dutil É, Mercier L (1994). Validation of the box and block test as a measure of dexterity of elderly people: reliability, validity, and norms studies. Arch Phys Med Rehabil.

[3] Liang KJ, Chen HL, Shieh JY, Wang TN (2021). Measurement properties of the box and block test in children with unilateral cerebral palsy. Sci Rep.

[4] Everard G, Otmane-Tolba Y, Rosselli Z, Pellissier T, Ajana K, Dehem S, Auvinet E, Edwards MG, Lebleu J, Lejeune T (2022). Concurrent validity of an immersive virtual reality version of the box and block test to assess manual dexterity among patients with stroke. J Neuroeng Rehabil.

[5] Villa-Berges E, Soriano AAL, Lucha-López O, Tricas-Moreno JM, Hernández-Secorún M, Gómez-Martínez M, Hidalgo-García C (2023). Motor imagery and mental practice in the subacute and chronic phases in upper limb rehabilitation after stroke: a systematic review. Occup Ther Int.

[6] Huertas-Hoyas E, Máximo-Bocanegra N, Diaz-Toro C, Montes-Diez R, Pérez-Corrales J, Sánchez-Herrera-Baeza P, Martínez-Piédrola RMª, García-Bravo C, Sánchez-Camarero C, Pérez-de-Heredia-Torres M (2020). A descriptive cross-sectional study of manipulative dexterity on different subtypes of multiple sclerosis. Occup Ther Int.

[7] Zapata-Figueroa V, Ortiz-Corredor F (2022). Assessment of manual abilities using the box and block test in children with bilateral cerebral palsy. Occup Ther Int.

[8] Mathiowetz V, Federman S, Wiemer D (1985). Box and block test of manual dexterity: norms for 6-19 year olds. Can J Occup Ther.

[9] Mathiowetz V, Volland G, Kashman N, Weber K (1985). Adult norms for the box and block test of manual dexterity. Am J Occup Ther.

[10] Hsiao CP, Zhao C, Do EYL (2013). The digital box and block test automating traditional post-stroke rehabilitation assessment. https://ieeexplore.ieee.org/document/6529516.

[11] Zhang Y, Chen Y, Yu H, Lv Z, Shang P, Ouyang Y, Yang X, Lu W (2019). Wearable sensors based automatic box and block test system. 2019 IEEE SmartWorld, Ubiquitous Intelligence & Computing, Advanced & Trusted Computing, Scalable Computing & Communications, Cloud & Big Data Computing, Internet of People and Smart City Innovation (SmartWorld/SCALCOM/UIC/ATC/CBDCom/IOP/SCI).

[12] Lee TKM, Lim JG, Leo KH, Sanei S (2018). Indications of neural disorder through automated assessment of the Box and Block Test. International Conference on Digital Signal Processing (DSP).

[13] Hashim NA, Razak NAA, Gholizadeh H, Osman NAA (2021). Video game-based rehabilitation approach for individuals who have undergone upper limb amputation: case-control study. JMIR Serious Games.

[14] Cho S, Kim WS, Paik NJ, Bang H (2016). Upper-limb function assessment using VBBTs for stroke patients. IEEE Comput Graph Appl.

[15] Oña ED, Jardón A, Cuesta-Gómez A, Sánchez-Herrera-Baeza P, Cano-de-la-Cuerda R, Balaguer C (2020). Validity of a fully-immersive VR-based version of the box and blocks test for upper limb function assessment in Parkinson's disease. Sensors (Basel).

[16] Oña ED, García JA, Raffe W, Jardón A, Balaguer C (2019). Assessment of manual dexterity in VR: towards a fully automated version of the box and blocks test. Stud Health Technol Inform.

[17] Gagnier JJ, Lai J, Mokkink LB, Terwee CB (2021). COSMIN reporting guideline for studies on measurement properties of patient-reported outcome measures. Qual Life Res.

[18] Tobler-Ammann BC, de Bruin ED, Fluet M, Lambercy O, de Bie RA, Knols RH (2016). Concurrent validity and test-retest reliability of the virtual peg insertion test to quantify upper limb function in patients with chronic stroke. J Neuroeng Rehabil.

[19] Plichta S, Kelvin E (2013). Munro's Statistical Methods for Health Care Research. 6th Edition.

[20] De Vet HCW, Terwee CB, Mokkink LB, Knol DL (2011). Measurement in Medicine: A Practical Guide.

[21] Bland JM, Altman DG (1986). Statistical methods for assessing agreement between two methods of clinical measurement. Lancet.

[22] Giavarina D (2015). Understanding bland altman analysis. Biochem Med (Zagreb).

[23] Koo TK, Li MY (2016). A guideline of selecting and reporting intraclass correlation coefficients for reliability research. J Chiropr Med.

[24] Haley SM, Fragala-Pinkham MA (2006). Interpreting change scores of tests and measures used in physical therapy. Phys Ther.

[25] Chen HM, Chen CC, Hsueh IP, Huang SL, Hsieh CL (2009). Test-retest reproducibility and smallest real difference of 5 hand function tests in patients with stroke. Neurorehabil Neural Repair.

[26] (2022). Validation of two digitized assessments for measuring hand function. OSF.

[27] Shrout PE, Fleiss JL (1979). Intraclass correlations: uses in assessing rater reliability. Psychol Bull.

[28] Hashim NA, Razak NAA, Osman NAA (2021). Comparison of conventional and virtual reality box and blocks tests in upper limb amputees: a case-control study. IEEE Access.

[29] Oña ED, Cuesta-Gomez A, Garcia JA, Raffe W, Sanchez-Herrera P, Cano-de-la-Cuerda R, Jardón A (2019). Evaluating A VR-based box and blocks test for automatic assessment of manual dexterity: a preliminary study in Parkinson's disease. https://ieeexplore.ieee.org/document/8882472.

[30] Portney LG, Dean EF (2020). Foundations of Clinical Research. Applications to Evidence-Based Practice. 4th Edition.

[31] Platz T, Pedersen AL, Bobe S (2023). Feasibility, coverage, and inter-rater reliability of the assessment of therapeutic interaction by a humanoid robot providing arm rehabilitation to stroke survivors using the instrument THER-I-ACT. Front Robot AI.

[32] Schädler S, Kool J, Lüthi H (2012). Assessments in der Rehabilitation: Band 1: Neurologie. 3rd Edition.

